# Enamel Tubules and Spindles: Enter and Exit the Amelocyte

**DOI:** 10.1007/s00223-026-01522-w

**Published:** 2026-05-09

**Authors:** Alan Boyde, David Mills

**Affiliations:** Dental Physical Sciences, Institute of Dentistry, Queen Mary University of London, Mile End Campus, London, E1 4NS UK

**Keywords:** Ameloblast, Kionoblast, Amelocyte lacuna, Microscopy, Neonatal cell death

## Abstract

By the commonly accepted rules of nomenclature, cells forming hard tissue which become incorporated within the inter- and extra-cellular tissue mass which they secrete are called ‘-cytes’: the spaces in which these cells sit are called lacunae. Hence, osteoblasts become osteocytes in osteocytic lacunae, chondroblasts become chondrocytes, cementoblasts cementocytes, even odontoblasts become odontocytes in some mammalian species. We provide evidence that enamel spindles are cell bodies of ameloblasts which have become entrapped and buried in the enamel matrix produced by the surrounding cells, to produce amelocytic lacunae: it is no life for a cell in a rigid, dense and dead tissue, and we do not suppose that amelo*cytes* live once they are set on that route. Spindles, normally only found at the enamel-dentine junction, are absent when the enamel at the junction is free of prisms, i.e., the ameloblasts had no Tomes’ processes. Spindles have an organic content, the amelocyte residue which can be stained - and removed by bleach, after which the spindle space no longer stains. Spindles are extensions of enamel tubules which result from the extension of a fine ameloblastic process [from the major Tomes’ process] which unites with an odontoblast process. In marsupial mammals, the spindle-like ends of the enamel tubules are commonly located well within the enamel. Spindles/amelocytes may form at the neonatal line in human deciduous teeth in cases of severe neonatal stress, and these features simply represent encapsulation of moribund ameloblasts. The frequency of spindles at the enamel dentine junction is greater in cases where the tooth germ is severely deformed by growth pressure from the forming root of a neighbouring tooth.

**Supplementary Information:**

The online version contains supplementary material available at 10.1007/s00223-026-01522-w.

## Introduction

*Dentine tubules* are spaces left in calcified collagenous dentine matrix originally occupied by odontoblastic cell processes.

Likewise, *enamel tubules* are spaces left in calcified enamel organic matrix which were originally occupied by extensions of the secretory pole, ‘Tomes’s’ processes of ameloblasts [1–4, 6–8, 11, 12, 16, 20–22, 24–34, 36–39]. Although—with the exception of the wombat [38]—enamel tubules are very numerous and obvious features in the marsupial mammals and insectivores [11], they receive rather scant attention in the general enamel literature, probably because it is heavily biassed to studies of human teeth. However, one purpose of the present paper will be to assert that enamel tubules are abundant - but just not so obvious—in human and other placental mammalian teeth.

Of *spindles*, Charles S Tomes wrote in his textbook [37, p. 29] ‘Cavities, or at least what look like cavities of irregular form sometimes exist in the enamel close to the surface of the dentine and when such space exist the tubes sometimes communicate with them; perhaps these are to be regarded as abnormal, but they are not uncommon and disposed with some regularity. Römer says that they have organic contents in the fresh state and suggests that they are nerve endings. Bödecker, of course, regards them as filled up by protoplasm. Wahlkoff considers that prior to enamel deposition, the surface of the dentine is absorbed and that they are dentine tubes left unabsorbed’. On page 30–33 Tomes continued… ’dentine tubes may occasionally be seen to enter the enamel, passing across the boundary between the two tissues, and pursuing their course without being lost in irregular cavities, though this appearance is seldom to be found. As was first pointed out by my father, the passage of tubes continuous with the dentinal tubes into and through a great part of the thickness of the enamel takes place in marsupials which such constancy as to be almost a class characteristic’. Tomes’ one figure of spindles (Fig. 9, page 30) is captioned ‘Cavities in human enamel which communicate with the dentinal tubes’. Of marsupial enamel ‘tubes’, he was ‘of the opinion that the tubes are in the substance of the prisms’ (p. 510). 

Meyer ([23], translation by Churchill) had excellent photomicrographs of spindles and stained them with fuchsin. He captioned them as ‘dentinal fibrils in the enamel’ or ‘club-shaped processes of the dentinal fibrils in the enamel’ (pp 67–69). ‘As the extensions of the odontoblasts are not passively enclosed in the enamel, they must have actively grown in between the ameloblasts during amelogenesis’.

The classic British English textbook Scott and Symons [31] stated ‘In some areas dentinal tubules pass into the enamel for short distances and are surrounded by the interprismatic substance of the enamel. They may terminate as pointed or rounded processes or may have a noticeably thickened end: in the latter case, they are known as enamel spindles. They are found in greatest numbers in the regions of the cusps’—‘The enamel spindles must presumably be produced by odontoblast processes which insinuate themselves between the cells of the internal enamel epithelium of the enamel organ before either dentine or enamel is laid down. It has been suggested that the enamel spindles do not strictly enter the enamel, but are exaggerated forms of the projections which alternate with the bays of the enamel dentine junction’. Recognising tubular enamel (p. 288) in marsupials, primates and insectivores, they wrote: ‘The enamel spindles of human teeth probably represent a limited form of tubular enamel, for all degrees of tube penetration, from the small localised spindles to the well-marked tubes characteristic of the enamel of Marsupials have been described within the Primate order.’

The classic American English textbook, Orban’s Oral Histology and Embryology, carried the same excellent figure and caption through many editions, distinguishing enamel spindles ‘from odontoblastic process in dentin’ [1, 30]. 

Schmidt and Keil ([29]: translated by Poole and Darling) considered spindles as Tomes’ fibres (i.e., odontoblastic cell processes) or dentine tubules in enamel. ‘Often not only the main branch of the dentine tubule enters the enamel but strongly developed lateral branches as well enter the enamel.’—‘if the ends of the tubules in the enamel widen, they become “spindles”’.—‘The sharp boundaries of the spindles—with convexities turned outwards—demonstrate that the spindles are formed by resorptive expansion of dentine canals entered in dentine probably under the influence of the Tomes’ fibres. The lumina of the spindles perforate the neighbouring prisms ([29], page 383; see also [17], page 51 ‘Kolbenförmige Fortsȁtze der Dentinkanȁlchen in den Schmelz’).

In summary, the literature is unanimous in concluding that both enamel tubules and spindles are extensions of odontoblastic processes in enamel. To counter this view, the aim of this paper is to present our recent discovery of spindles in the middle of enamel and examine evidence that spindles are spaces generated by dead ameloblasts.

## Materials

### Marsupials

Mature teeth from dried skulls *Trichosurus vulpecula*,* Phascolarctos cinereus*,* Dasyurus maculatus*,* Macropus rufus*,* Rufous betong*,* Wallabia bicolor*,* Macropus rufogriseus* [8]. Developing teeth obtained from pouch young of *Didelphis nudicaudata* [7, 8] and *Trichosurus vulpecula* [20]. Fossil *Diprotodon* tooth.

### Human and Other Mammals

A collection of ground sections of human teeth (incisors, canines, premolars and molars) and other mammalian teeth assembled by AB from 1959 onwards and all prepared from teeth obtained prior to the UK Human Tissues Act 2004, therefore no ethical issues. Two collections of ground sections of shed human deciduous teeth assembled by: (1) Dr Janice M. Fearne from low-birth-weight (LBW) children as part of her (LHMC, QMUL) PhD 1995 [14, 15] and: (2) the late Professor Aubrey Sheiham from 1960s cases of severe nutritional deprivation in Nigeria.

### Methods

- *Fracturing* to view the enamel tubules and dentine tubules at the enamel dentine junction (EDJ) using 3D scanning electron microscopy (SEM).
- *Embedding* in methyl methacrylate monomer polymerised to poly-methyl-methacrylate (PMMA).
- *Polishing* block surfaces on graded silicon carbide abrasive papers, wet, to 4000 grit: backscattered electron mode SEM (BSE-SEM).
- *Etching* with acids to expose methacrylate casts of tubules and spindles where the infusion of monomer had been successful: SEM.
  - Followed by removal of demineralised dentine matrix with 5% available chlorine sodium hypochlorite solution: SEM.
- *Airpolishing* with wet sodium bicarbonate powder in a water jet [8].
  - Prior removal of dentine organic matrix by treatment with sodium peroxide: SEM.
- *Scanning electron microscopy*
  - Cambridge Stereoscan S4-10 and Zeiss DSM962.
    - Gold coated samples 10 kV SE.
    - Carbon coated samples 20 kV BSE-SEM [5].
  - Zeiss Evo MA10, uncoated samples, 50 Pa chamber pressure, 20 kV BSE-SEM.
    - *High pressure mercury porosimetry*: human teeth subjected to mercury intrusion at very high pressure: embedded PMMA, cut, polished, 30 kV BSE-SEM.
- *Light microscopy (LM) of ground sections*:
  - Ordinary transmitted light microscopy.
    - Through focus series.
  - Linearly polarised light microscopy (LPL, PLM).
  - Half wave retardation plate—sensitive tint.
  - Multiple rotation linear polarised light microscopy, computer control.
  - Confocal scanning light microscopy in reflection mode, Tracor TSM.
    - Through focus series.
  - Dynamic aperture microscopy (DAM): multiple oblique illumination series to generate 3D views.
  - Giemsa staining of PMMA block surfaces.
- *Transmission electron microscopy (TEM)*: of ultra-microtomed sections of methacrylate embedded tooth germs of pouch young *Didelphis nudicaudata* [8, [7]: Siemens Elmiskop I, 80 kV: 3D imaging by tilting.
- *Oxygen plasma ashing*: to remove methacrylate embedding resin to expose the developing enamel surface: SEM.
- *Image processing software*: Paint Shop Pro 5; ImageJ-Fiji; Syncroscopy Cambridge AutoMontage for through focus stack processing; AmScope for LM image acquisition and stack processing.
- *X-ray microtomography (XMT)*: QMUL MuCat system [13].

## Results

**Fig. 1:**
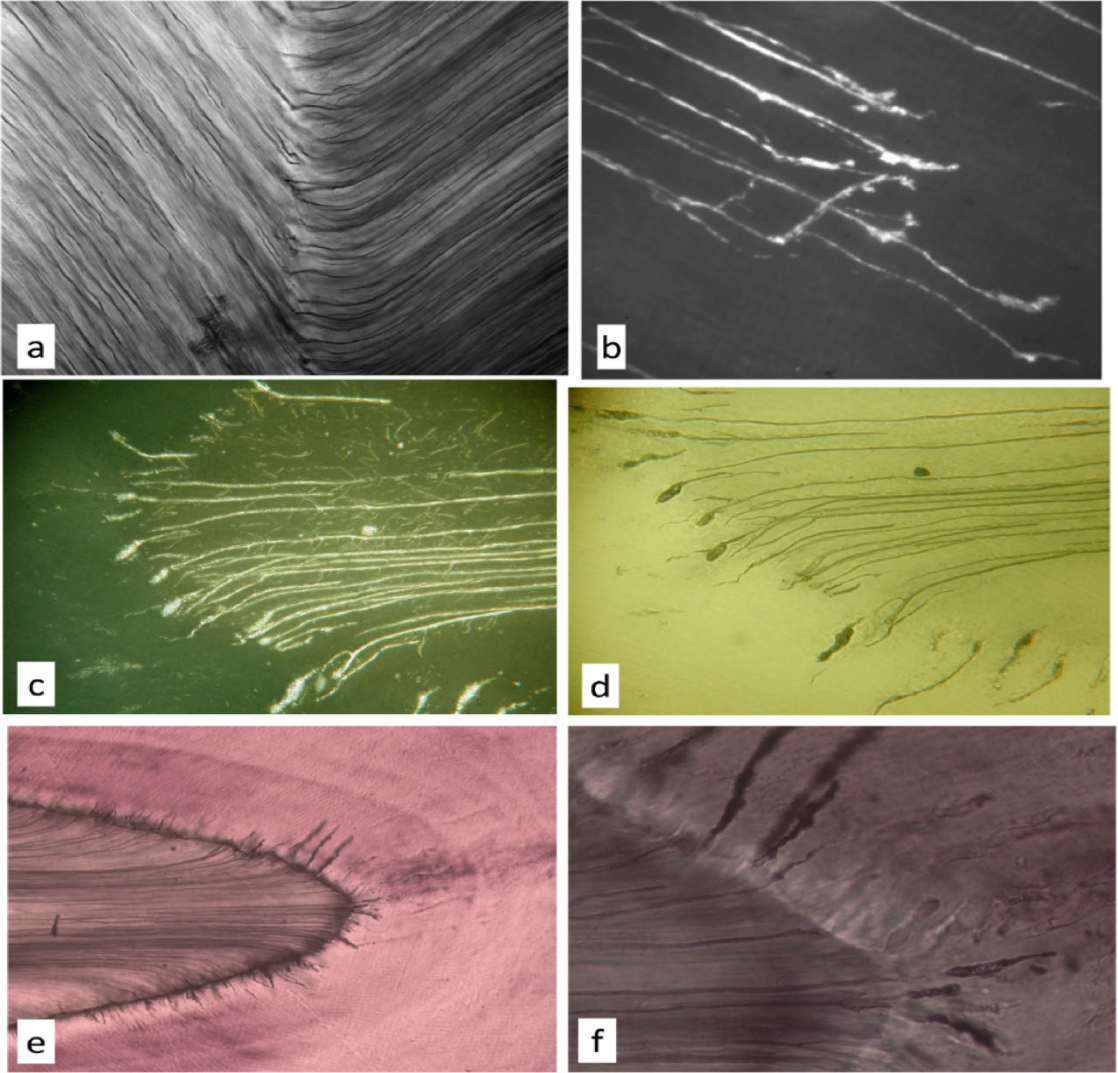
Light microscopy of tubules and spindles. **a** Ground section of wallaby *Notamacropus rufogriseus*, a recent macropod marsupial, molar, EDJ, enamel left, showing continuity of enamel tubules and dentine tubules, LM with linear polarising filters uncrossed by 15°. Field width 295 μm. **b** Cut and polished surface of enamel in *Diprotodon*, a giant fossil macropod marsupial, showing enlarged endings of enamel tubules in mid-thickness enamel, confocal spinning disc reflected light image using Tracor TSM—sample courtesy of Keith Lester, Sydney. Field width 200 μm. **c** LS human premolar near cusp tip, TSM reflected light through focus stack processed with AutoMontage software, showing continuity of dentine tubules and enamel spindles at EDJ. Field width 250 μm. **d** same sample, through focus *transmitted* light stack processed with AutoMontage. Field width 250 μm. **e** LS human incisor cusp tip, oblique transmitted light one frame from a ‘DAM’ movie. The complete sequence giving a 3D image can be seen in Supplementary Figures. Field width 500 μm. **f** same, Field width 250 μm. Movie in Supplementary Figures

**Fig. 2:**
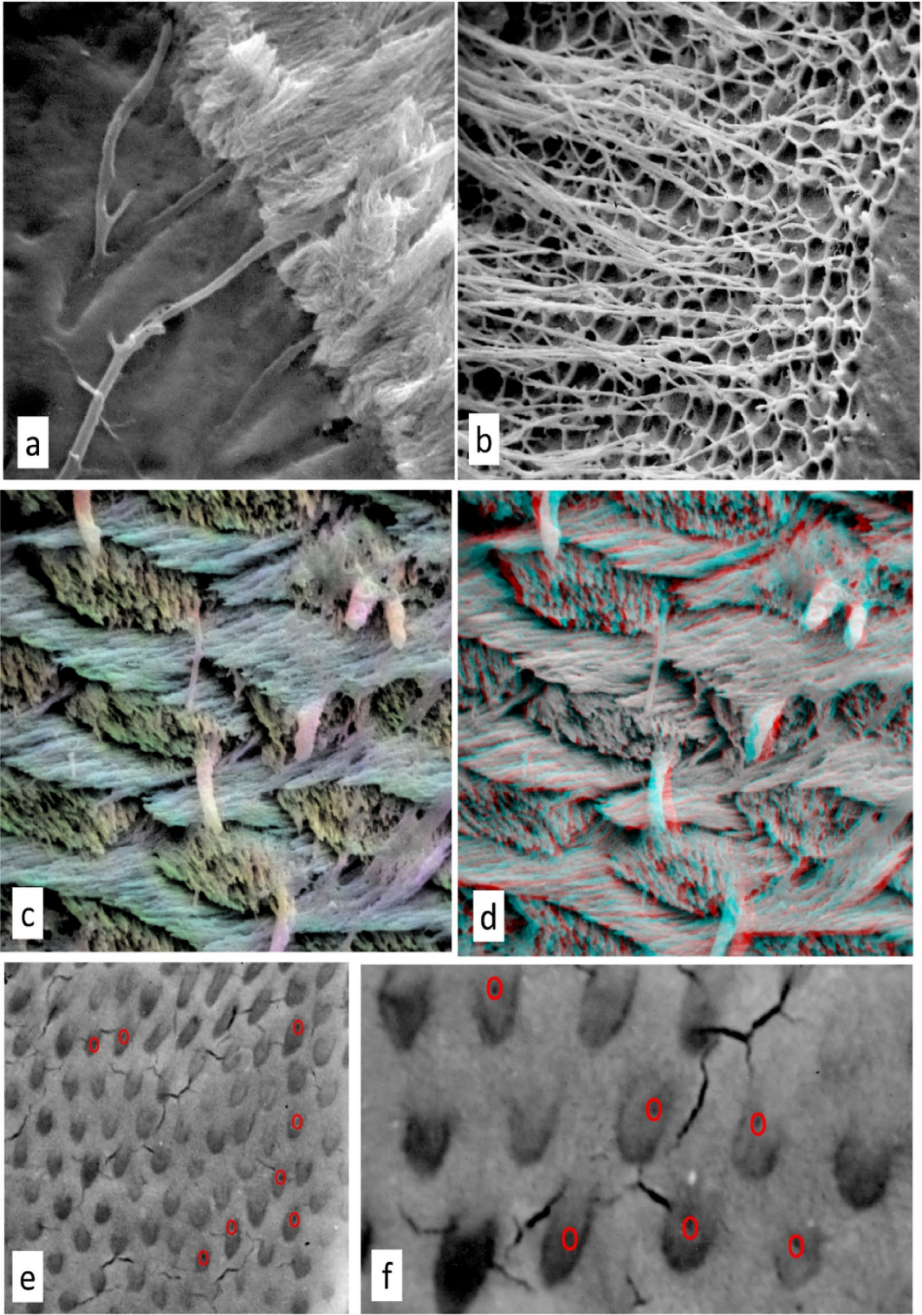
SEM images of marsupial enamel tubule casts and spaces. **a** *Trichosurus vulpecula*, brushtail possum, molar embedded in PMMA, polished LS cut surface etched 2 min with M HCl to show the continuity of the dentine (left) tubules with those in enamel (right). Field width 18 μm. **b** *Trichosurus vulpecula* molar embedded in PMMA, etching for 2 min with M HCl has removed enamel to a considerable depth, exposing many layers and lengths of enamel tubules which have clumped together in the drying process. Field width 83 μm. **c** *Dasyurus maculatus*, quoll, molar embedded PMMA etched 0.1 M HCl for 1 min, freeze-dried, showing tubule casts located within Pattern 2 enamel prisms. One prism has 2 tubule casts. Colour coding for orientation using ImageJ OrientationJ. Field width 18 μm. **d** Same field, anaglyph stereopair tilt angle difference 10, for viewing with red-cyan filters. **e**, **f** Didelphis nudicaudata, rat-tailed opossum molar TEM block embedded PMMA, oxygen plasma ashed to expose Tomes’ process pits at 4.2 μm intervals in mineralising front surface of developing enamel: location of tubule openings ringed in red

**Fig. 3:**
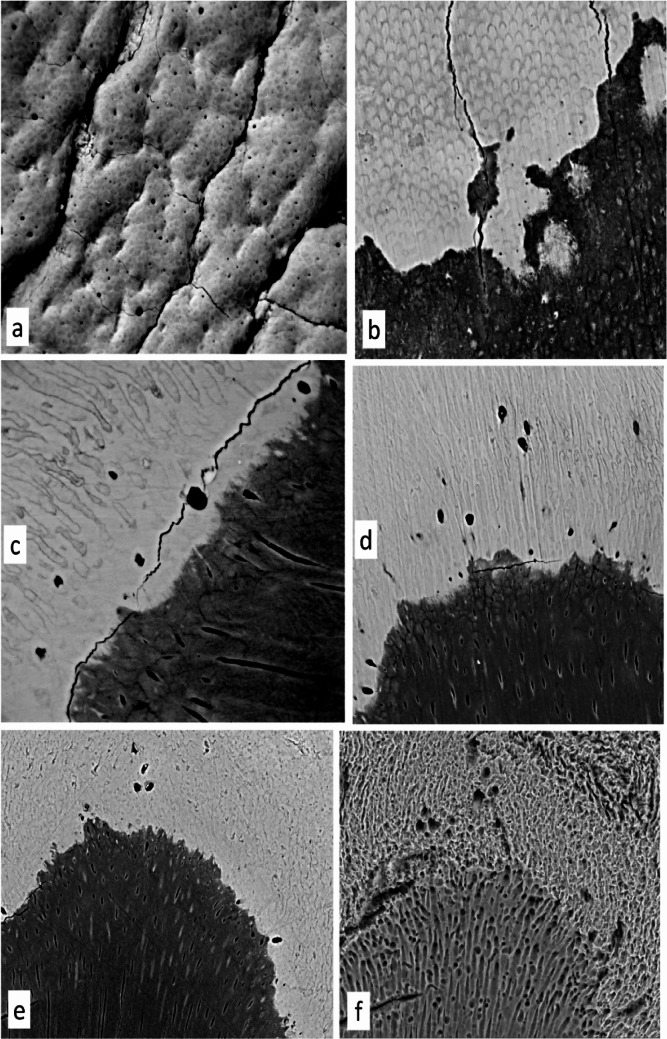
SEM images of spindle space at EDJ in human permanent molars. **a** Enamel side of EDJ in human molar exposed by first making the dentine anorganic by prolonged treatment with 50°C 1% sodium peroxide solution followed by AirPolishing using jet of sodium bicarbonate power in air surrounded by water. Larger black spaces are enamel spindles, smaller enamel tubules. Cracks develop in the enamel due to the removal of its dentine support. Field width 200 μm. **b** Human permanent molar [Amelogenesis imperfecta case] embedded PMMA, polished surface at tangent to EDJ, showing small black spaces in enamel which are enamel tubules. Field width 137 μm. **c** Lower third permanent molar embedded PMMA, polished surface at EDJ, larger dark spaces in enamel are spindles. Field width 90 μm. **d** Molar, PMMA, micromilled surface at EDJ, larger dark spaces in enamel are spindles. Field width 178 μm. **e** Molar, included rather than embedded in PMMA, polished surface at EDJ, dark spaces in enamel are spindles. Field height = 196 μm. **f** Same region as **e** after etching with 16% HCl ‘Super etch’ gel for 1 min, followed by 5% available chlorine NaOCl solution to remove demineralised dentine matrix: showing considerable expansion of spindle spaces in the enamel. Field height = 213 μm

**Fig. 4:**
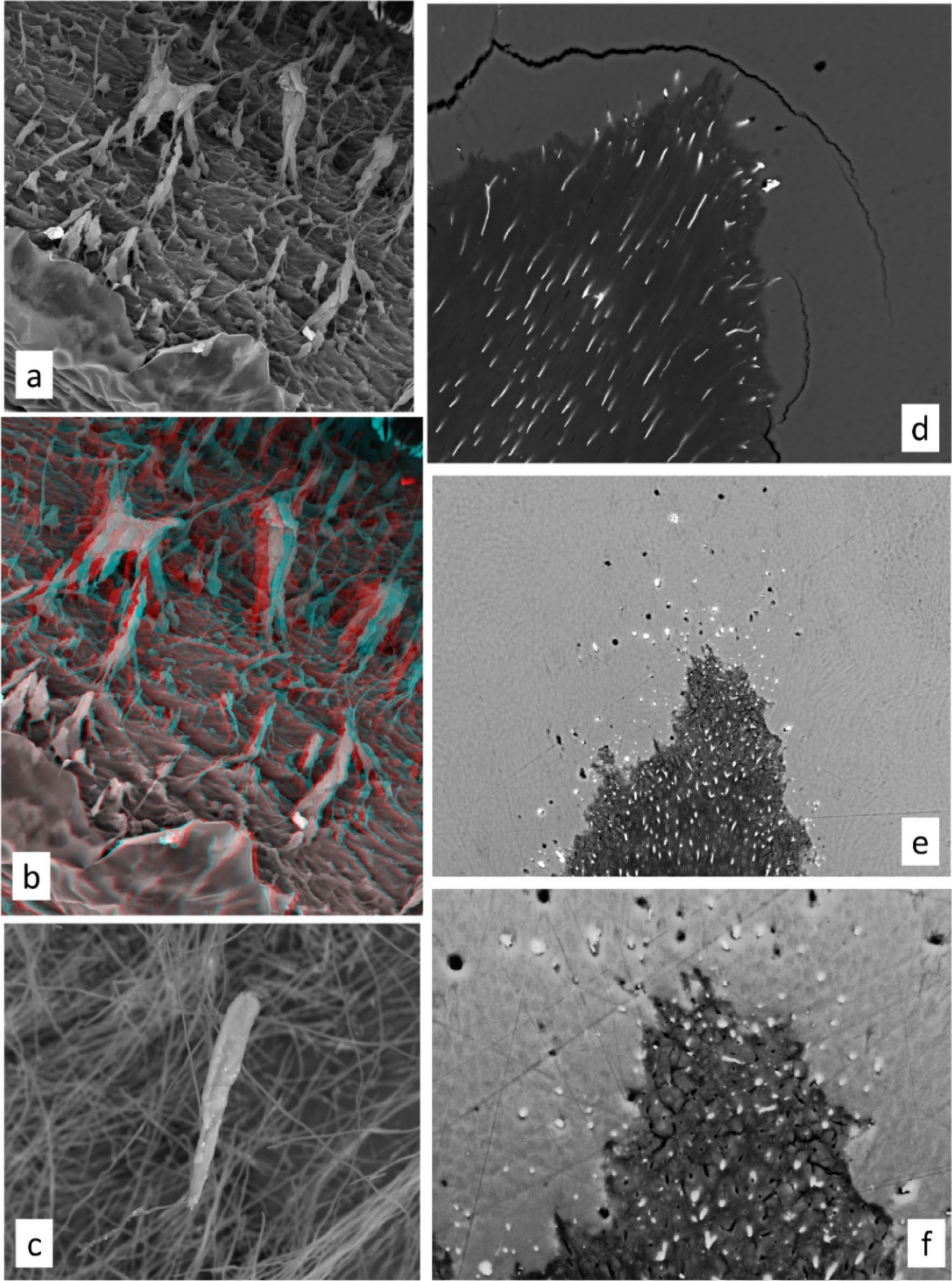
SEM images of spindle and tubule casts, and Hg porosimetry. **a**, **b** PMMA embedded LS human premolar, polished, enamel dissolved in M HCl, washed, freeze-dried, showing casts of enamel tubules and spindles and tuft planes. Field width ~ 160 μm. **b** Anaglyph stereo-pair image, view with red-cyan filters. **c** PMMA cast of spindle nested on a bed of casts of dentine tubules. 2 M HCl demineralisation followed NaOCl bleach removes all dentine, so features move from original positions. Field height 155 μm. **d** 30 kV BSE SEM image of Hg porosimetry sample, showing continuity of dentine and enamel tubules and spindles. Lower right first premolar, lingual cusp tip, EDJ: direction of entry of Hg filled tubules can be seen from the greater blurring and loss of signal intensity with depth into the surface. Field height 240 μm. **e**, **f** 30 kV BSE SEM image of Hg sample, showing tubules and spindles. Lower left first premolar, buccal cusp tip. Some spindle spaces are dark because they are empty. **e** Field height 384 μm. **f** Field height 146 μm

**Fig. 5:**
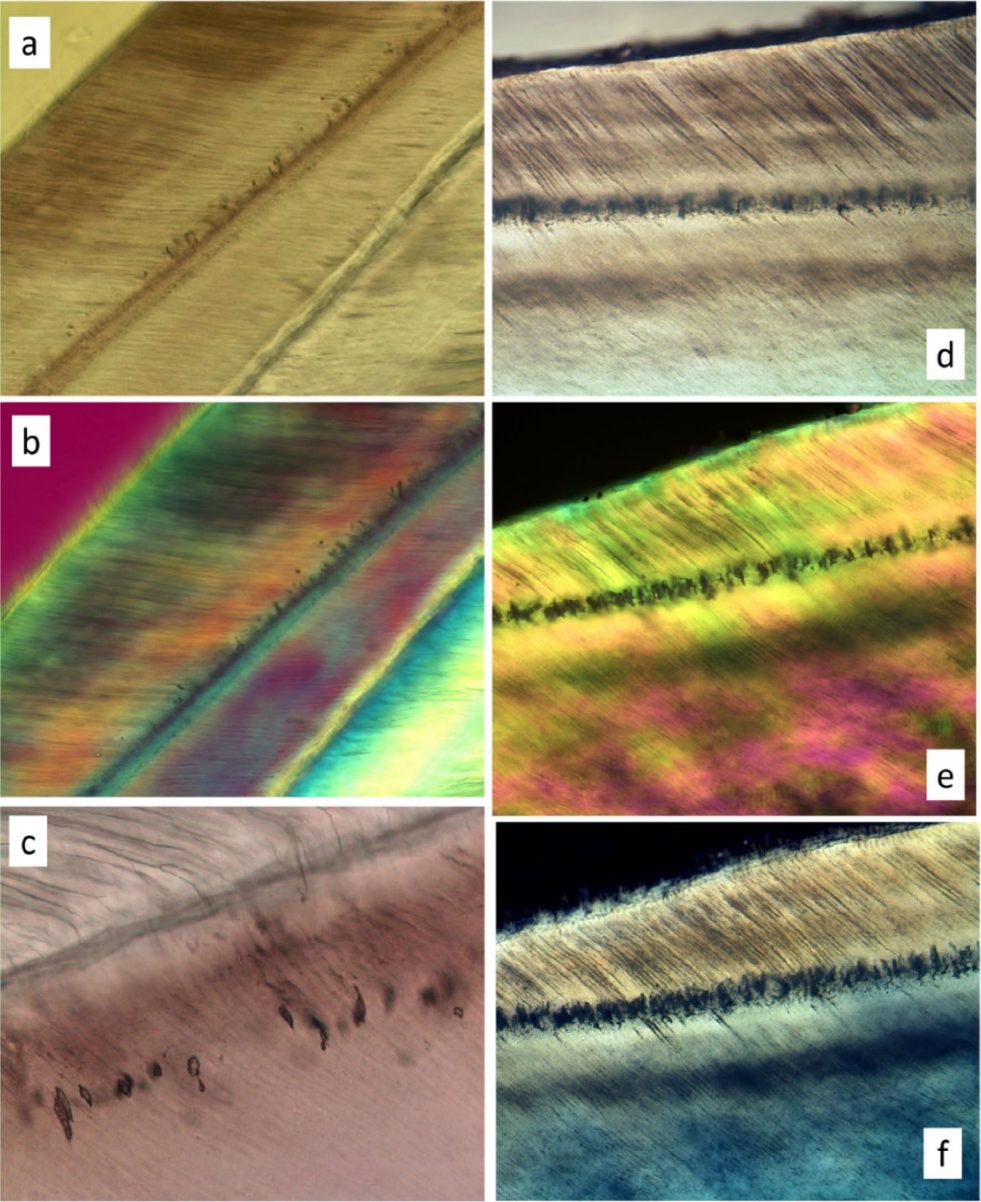
LM of ‘spindles’ at neo-natal line in (**a**–**c**) low birth weight and (**d**–**f**) poor nutritional status deciduous teeth. **a**, **b** Lower central deciduous incisor, bucco-lingual LS, lingual face: Janice Fearne LBW case 602. Dark features perpendicular to the NNL are interpreted as spaces left by incorporation of dead ameloblasts. Both are single frames from ‘DAM’ movies, field widths 570 µm. **a** Ordinary oblique transmitted light: **b** crossed linear polarising filters with a half-wave plate sensitive tint filter. **c** Upper lateral deciduous incisor, bucco-palatal LS, palatal face: dentine top: Janice Fearne LBW case 529. Dark spaces included in enamel - interpreted as spaces left by incorporation of dead ameloblasts - lie outside the plane of the neonatal line which is not itself clearly marked. Through-focus stack processed with AutoMontage software to produce extended depth of field image. Field width 295 µm. **d**–**f** Aubrey Sheiham Nigerian malnutrition case 11-3b. Upper central deciduous incisor, bucco-palatal LS, field widths 570 µm. All show dark spaces interpreted as spaces left by incorporation of dead ameloblasts. **d** ordinary oblique transmitted light, single frame from ‘DAM’ movie. **e** circularly polarised light, AmScope software extended depth of field from through focus stack, maximum contrast option. **f** minimum intensity through 69 µm focus PLM image stack

**Fig. 6:**
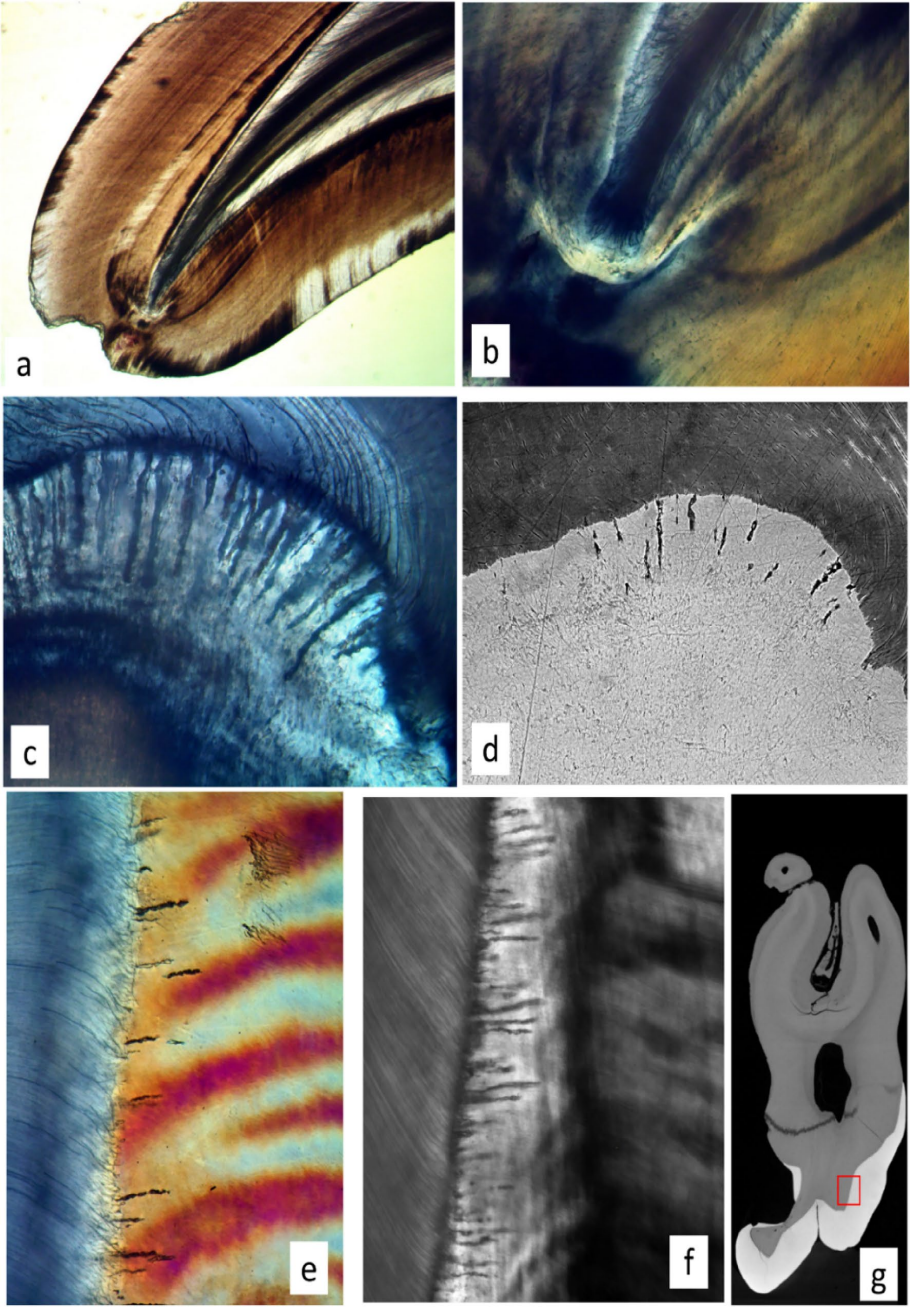
No prisms > no spindles. Deformation of EDJ > more spindles. **a**, **b** Bucco-palatal longitudinal ground section of upper lateral incisor with a prism-free enamel patch at the incisal edge. There are no enamel spindles in the prism free region, but they appear where the enamel becomes prismatic further from the biting edge. **a** ordinary transmitted light, field width 2950 μm. **b** circularly polarised light, field width 570 μm. **c**, **d** Mesio-distal longitudinal ground section of upper third molar showing location of a fold in the occlusal EDJ with a dense patch of spindles in the depression. **c.** Circularly polarised light. Extended depth of field from 16 μm through focus stack. Field width = 295 μm: **d** BSE SEM of same field. **e** Mesio-distal longitudinal ground section of distal EDJ in upper third molar in which the mesial surface of the tooth germ was impacted by the growing disto-buccal root of the second molar during development, showing a dense array of enamel spindles in the mid lateral enamel on the distal side. Extended depth of field from 42 μm through focus stack. Field height 570 μm. **f** Another mesio-distally compressed upper third molar (AB’s own), similar location in LS, CPL, also showing numbers of spindles where they are not found in normal teeth. Single focus. Field height 570 μm. **g** Single tomographic slice from the same tooth as **f** showing the mesio-distal compression. The red box shows the location of the physical LS shown in **e**, **f**

Enamel tubules and spindles are shown in Figs. 1, 2, 3, 4, 5 and 6(a–f): they are continuous with dentine tubules, as evidenced by conventional LM and reflection confocal LM and SEM of fractured or embedded and polished surfaces [7, 8], and SEM of resin casts (Fig. 2a–d). The spindle is an expansion of an enamel tubule (Fig. 1c–f). This is clearest in the case of marsupial enamels where the bulb- or spindle-like expansion and the end of the enamel tubule lies at a distance from the EDJ (Fig. 1b). Spindles stand perpendicular to the incremental plane, i.e., to the developing enamel surface which was in force at the time. They are usually not, therefore, either parallel with prisms or at right angles to the EDJ. This means that spindles have the same orientation as would have had the ameloblasts which stand more or less perpendicular to the developing enamel surface, whereas, because ameloblasts move across that surface, the prisms—the traces of ameloblastic movement with time—are not quite perpendicular.

The locations of tubule entrances at the developing enamel surface in marsupials can be seen in oxygen plasma ashed preparations of PMMA embedded TEM blocks and are within the Tomes’ process pits, i.e., within the prism body—not in a possible inter-row sheet, interameloblastic, interprismatic location (Fig. 2e, f). That they are in prisms or at the prism boundary discontinuity can also be seen in part-casts (Fig. 2c, d).

Although short, enamel tubules are common in human teeth. The smallest of human enamel tubules lie near the limit of resolution of conventional optical microscopy for thin samples, and ground sections of enamel are not thin. These narrower, blind-ending enamel tubules are much shorter than ameloblasts or spindles, and an artificial prepared surface (e.g., a polished block face for BSE SEM) is unlikely to intercept many of them—either tangentially because they are so short or longitudinally because they are not densely distributed. The ideal would be to have a preparation in which the enamel side of the EDJ was exposed following its own 3D contour. The best approach that we have so far used is to remove all the dentine by first making it very soft by removing the organic matrix by treatment with a hot sodium peroxide solution, followed by hosing the dentine away with a water jet as in AirPolishing [8; Fig. 3a]. This treatment, albeit that a little enamel may also have been lost, gives rise to surfaces which show a more abundant set of features which we identify as tubules and spindles as well as the ubiquitous, less densely mineralised tuft planes. The method is difficult to apply in the constrained space previously occupied by dentine in the narrow tips of cusps where spindles are at their most frequent.

However, all polished block surfaces viewed by BSE-SEM—whether or not properly embedded in PMMA or another resin—demonstrate fine dark profiles from circles to elongated ellipses which are in the same size range as the features which are unquestionably dentine tubules in the same preparations (Fig. 3b–e). The finest dentine tubules close to the EDJ are sometimes obliterated by the higher density (white in BSE-SEM) peritubular dentine phase (Fig. 3c–e).

Larger dark spaces are common in those regions known to have the highest frequency of spindles and we recognise them as such (Fig. 3a–e). Their diameters are up to those of prisms, i.e., in the same size range as ameloblasts.

In trying to produce PMMA casts of the largely inaccessible enamel tubule and spindle spaces in an intact tooth, a main difficulty is to ensure the removal and replacement of water by an intermediate solvent series (ethanol, xylene) and thus the proper substitution and filling by the methacrylate monomer prior to polymerisation. To increase chances of success, it is better to start with thin samples, for example, standard ground sections. Removal of enamel mineral—which is most of enamel—will instantly reveal whether one has been successful with embedding. Otherwise, when dissolving/etching enamel with acids, enamel tubules and spindles, both being spaces in enamel, are rapidly expanded, enamel tubules beyond recognition, and the spindles grossly enlarged (Fig. 3f). Leaving the adjacent demineralised dentine matrix in place keeps the tubule and spindles casts in place (Figs. 2a, b and 4a, b). Tubules and/or spindles without the supporting matrix in which they existed can simply float around (Figs. 2b, 4c: see also Meyer’s [23] Fig. 63, page 58 of ‘Dentinal fibrils, partly isolated, fuchsin staining’).

Tubules and spindles are, effectively, relatively large empty spaces, permeable to water and to dyes, other solvents like ethanol, xylene and methacrylate monomer, the latter enabling us to make PMMA casts. Spindles are filled with air in dry samples. Mercury penetrates and is retained after high pressure mercury porosimetry testing in any significant spaces in enamel, including the prism boundary discontinuity (sheath) space in hypomineralised enamel as well as tubules and spindles (Fig. 4d–f). Some spindle spaces lack mercury after prolonged storage or observation in the SEM, but this can be attributed to the surface tension and volatility of the metal in bulk. Thus, whilst observing such specimens in the SEM, balls of metal could be observed to move and disappear. BSE-SEM image sequences taken in quick succession show the live movement of mercury (Supplementary Figure).

We discovered spindles-like spaces at the level of the neo-natal line in two of the Janice Fearne [14] LBW series and one case from the Aubrey Sheiham Nigerian series of maternal malnutrition at the time of birth series of deciduous tooth ground section (Fig. 5a–f). These features, like spindles, are dark in transmitted light and bright in reflected light. They are elongated in a direction perpendicular to the neo-natal line—i.e. perpendicular to the developing enamel surface at the time of development—and not quite parallel to the prism direction, as is the case with spindles at the EDJ in general.

Neonatal hypoplasia of enamel was found in cases from both series. By LM, the enamel is missing after the neonatal line and the profile of the enamel surface shows the picket fence appearance due to retention of the Tomes’ process pits. The developing surface type of pit is seen by SEM [2, 4, 14]. The prenatal enamel proceeds to maturation with no further enamel secretion [15]. We cannot know whether, or how many, ameloblasts survived into the maturation stage, but if there is no recovery of secretory potential after the neonatal disturbance then there is no enamel in which to bury defunct, dying or dead ameloblasts.

We found a longitudinal bucco-palatal ground section through an upper permanent incisor which had non-prismatic enamel starting at the tip of the incisal edge. This means that the ameloblasts which started to form the enamel in this tooth had had no Tomes’ processes, since these are required to form prisms. This innermost region of the enamel also had no enamel spindles and no enamel tubules—no extensions of the dentinal tubules across the EDJ. Slightly further along the tooth, prismatic enamel starts - and includes spindles. No Tomes’ process = no spindles (Fig. 6a, b).

In a mesio-distal longitudinal ground section of an upper third molar we found a constellation of spindles associated with a fold in the EDJ. i.e., in a conformation opposite to that found at a cusp tip, incisal edge, or marginal ridge (Fig. 6c, d).

We studied two upper third molars which had been deformed by the impaction of the disto-buccal root of the second molar into the mesial surface of their tooth germs. XMT and BSE-SEM of these teeth showed severe deformation of the EDJ and absence of enamel over substantial regions of the depressed mesial surface of the tooth. In both cases, subsequently prepared longitudinal mesio-distal ground sections studied by LM showed an obvious dense content of spindles in the distal enamel, i.e., the side opposite to a mesial compacting pressure which we assume to have existed (Fig. 6e–g).

Spindles close to PMMA block surfaces were stained purple with Giemsa stain, the stain was removed by treatment with 5% available chlorine hypochlorite bleach, and, after washing the stain was not taken up in the spindle spaces.

## Discussion

Both recent and older textbooks are united in the belief that enamel *tubules* are extensions of odontoblastic cell processes into enamel, and further ascribe the same origin to enamel spindles, which are held to be expansions of such odontoblastic cell processes in enamel (Churchill-Meyer [23]). Enamel tubules and dentine tubules are indeed continuous at the enamel-dentine junction (EDJ). This arises from the fact that pre-ameloblasts and odontoblasts make some kind of permanent ‘handshake’ contact before either tissue is formed [20].

As regards *marsupials*, Lester [20] showed the presence of fine extensions of the Tomes’ processes into enamel connecting with processes from odontoblasts. Sasagawa and Ferguson, [27] could find no special junctions between these two cell types, but they are both represented by tubules and the tubules are continuous one with the other. Mostly SEM-based studies showed that most enamel tubules are continuous with dentine tubules at the EDJ. The most frequent location of enamel tubules is within the prism, or at prism boundary discontinuities. Not every prism has a tubule, but some have more than one. Tubule locations can be seen as deficiencies opening at the developing enamel surface. Enamel tubules may sometimes reach to the outer enamel surface and are easily identified in worn occlusal surfaces [8].

The one marsupial species in which enamel tubules are absent is the wombat. Lester and Boyde, [21] wrote that ‘There seems little doubt that the absence of *“enamel* tubules” in the wombat *(Vombatus ursinus)* is related to the existence of a well-developed mantle dentine layer. Thus, whereas the kangaroo *(Macropus sp.)* and opossum *(Metachirus nudicaudatus)* possess only a thin layer which might be called mantle dentine (with finer and less numerous yon Korff bundles oriented nearly parallel to the dentinal tubules where these reached the enamel-dentine junction to become continuous with the enamel tubules), the wombat has large and numerous yon Korff fibril bundles oriented parallel to the enamel-dentine junction. These bundles appear to preclude the close approach of the dentine tubules to this plane. We have frequently found in the placental mammals that areas of easily demarcated yon Korff fibrils alternate with areas lacking these but exhibiting enamel ‘tubules’ - enamel ‘tubules’ in placental mammals being more common than is generally realized’.

Enamel tubules are numerous in human and other mammalian enamels, but are so small that they are difficult to see by LM and are lost in etched preparations for SEM. We recognised the high frequency of small enamel tubules at the EDJ in human teeth when working with Keith S. Lester, with stereoscopic TEM imaging of replicas of fractured surfaces - in the interregnum between 1960, when aware of the great advantages of SEM, and 1964, an SEM being available for purchase and 1966 getting a grant to buy one. Unshadowed carbon replicas show only the morphology of the surface, whereas all SEM imaging modes are influenced to some considerable extent by the bulk properties of the substrate enamel or dentine, such that finest surface details may be obscured. The morphology shown in TEM of such replicas could only be seen in stereo, and most journal readers were (and still are, apparently) unwilling or unable to view 3D images, which have therefore been unacknowledged. Unfortunately, all our 1960s TEM plates have long since disappeared and we are no longer able to provide that evidence. However, short enamel tubules are very common in human teeth, and we, hypothesize, also represent the result of a connection between a thin connection of a thin process extending from Tomes’ process with a fine end process of an odontoblast, which is severed very early in enamel development—e.g. after a few days.

Not only wet acid etching increases enamel tubule diameter: in the first recorded SEM study of any hard tissue, we reported the increase in size of marsupial enamel tubules etched by 5 keV Ar^+^ ions in the SEM [33], and this was confirmed in TEM replica studies of the same samples (e.g., Figs. 5.7 and 5.8 in [2]; Fig. 22 in [7]).

A third class of feature near the EDJ that we should consider are the tuft planes [4]. Tufts are strips of hypomineralised enamel running in the longitudinal direction of a tooth which extend for a few hundreds of microns from the enamel-dentine junction, for example to a sixth or a quarter of the enamel thickness, depending on location. From the viewpoint of this paper, the important facts are that they are continuous sheets, a few prisms wide, and have an extent much greater than ameloblasts are long, and very extensive in the longitudinal direction. They are partly but poorly mineralised, not empty like spindles. They follow the decussating courses of the prisms—spindles do not. The enamel within tufts does not mature. They seem to arise as faulting planes during early maturation stages. They have not been identified at early developmental stages where the developing enamel is still thin and, as yet, immature. Their under-mineralisation is such that they always show in high contrast x-ray microtomography (µCT, XMT) of mature enamel and can still be seen with at voxel dimensions which do not resolve prisms, i.e., they are wider than prisms or ameloblasts. Spindles, on the other hand, cannot be resolved easily until the resolution is below 2 μm. We shall conclude that *spindles* in enamel are dead ameloblasts. As regards the death of a proportion of an ameloblast population, Saunders et al. [28] in the rat molar, Symons [35] in the rat incisor described a distinctive class of cells christened kionoblasts. Boyde [2, pp 80 & 81, Figs. 2.24.1 and 2.24.2] also figured kionoblasts in TEM studies of coypu incisor ameloblasts: they certainly look like sick and dying cells, but, to our knowledge, no such subpopulation has yet been identified in human tooth germs. Kreshover et al. [19] studied extensive series of tooth germs from neonatal human infant mortalities. They showed several pathological changes in ameloblasts but did not address the issue of instances of isolated cell death and inclusion in enamel matrix.

Spindles are concentrated at cusp tips. The time and the place to look for their development would be in the tips of the largest, first-formed cusps of first permanent molars since these have commenced amelogenesis before birth. Pre-term tooth germs should provide the scenario. Does any 20th century histology collection exist which might provide suitable study material?

The content(s) of tubules and spindles will originally have been cell membrane and cytoplasm. As such, solid matter would be minimal, with probably 85% volume fraction of water. The histological preservation of cell debris in enamel would certainly not be expected. We have shown that there is something in spindle spaces which can be stained (e.g., with Giemsa), that this can be removed by treatment with hypochlorite bleach and that the same stain will not demonstrate spindles afterwards—thus showing that the stain was not simply attaching to bare crystal surfaces.

The lines of evidence that suggest to us that spindles arise from ameloblasts are:

- The spindles have the same size and length and orientation as ameloblasts.
- In the absence of Tomes’ processes of the ameloblasts, no spindle is formed.
- Dying ameloblasts have been seen, albeit in other species.
- The end bulbs of the enamel tubules in marsupials have the same shape and form as ameloblasts, and are remote from odontoblasts. Mummery, [25] drew particular attention to the fact that the ‘bulb like’ terminal swellings of enamel tubules in marsupial enamel ‘have a great similarity to the so-called spindles in human teeth [see his Figs. 35, 36, 53 and 54]. Our TSM reflection mode microscopy of the giant fossil marsupial Diprotodon illustrates this point (Fig. 1b).
- In this paper, we identify spindles as occurring in the middle of enamel formation in human teeth at times of severe neonatal stress: such have no prior tubule in communication with dentine.
- Such stress is known to terminate secretory activity in a proportion of the ameloblast population, hence giving rise to ‘neonatal’ hypoplasia.
- The frequency of spindles increases locally where there is an unusual shape of the EDJ which might stress the ameloblasts.
- Equally the frequency of spindles is increased along relatively flat portions of the EDJ where it can be determined that the tooth germ was under compressive stress by impaction from an adjacent tooth germ.

We cannot concede or conceive that spindles and tubules present any functional advantage, other than speculating that there could be an advantage in opening dentine tubules prior to attrition reaching the dentine proper in order that odontoblasts might respond by forming a suitable secondary dentine barrier.

Why do spindles not mineralise? Of course there is space for mineral deposition, but there is no enamel matrix. Against this point, it might be argued that there is no bone matrix in dead osteocyte lacunae, yet they mineralise. In clear cases of apoptotic death in osteocytes, the characteristic apoptotic debris is heavily mineralised to produce micropetrotic pearls ([9, 18] and unpublished observations). At the same place and the same level as spindles, tufts also fail to mineralise, yet they have an abundance of enamel matrix protein [tuftelin]. By analogy with bone, bone which fails to mineralise on schedule in rickets and osteomalacia will never mineralise. Is there some kind of inhibition?

We are concluding that ameloblasts (amelocytes) die to become enamel spindles, but the exact mechanism of cell death remains to be determined, and we cannot assume that it would be apoptosis. Cell death in osteocytes is, morphologically, frequently apoptosis. Cell death from apoptosis is usually followed by the removal of cell debris by macrophages, but that cannot happen in bone, and of course it cannot happen in enamel.

Although the cell remnant situated in spindles is not to be expected to be well preserved and is extremely small in quantity, the notion that it is there at all should give rise to the possibility of looking for molecular fragments, including fragments of DNA in fossil and sub-fossil enamel. Techniques for the extraction of such information are burgeoning at the present time [10], but no one to date has thought of looking in enamel as such, and enamel is the best-preserved fossil tissue.

Finally, we additionally challenge the current concept that enamel tubules originate from odontoblastic processes crossing enamel dentine junction. There is no doubt that enamel tubules and the thin parts of spindles at the EDJ are continuous with dentine tubules which are occupied by single terminal branches of odontoblast processes (actually the beginnings of the odontoblast processes during development). We do not yet have the high-quality 3D TEM which would be required to see where the junction between the ameloblastic-enamel tubule-process and the odontoblast-end-processes meet in human teeth. We surmise that this juncture is in the enamel very near to the EDJ—Why would it be anywhere else? The possibilities are: (1) the odontoblast-end-processes lie between pre-ameloblasts before they differentiate and secrete enamel matrix-around these processes: however, the presumptive odontoblasts (PO) and pre-ameloblasts (PA) are in face to face contact before dentinogenesis and amelogenesis commence: (2) the odontoblasts push processes into enamel matrix –this just seems highly improbable, since it would require some kind of resorptive function: and (3) the contacts between POs and PAs persist after they start secreting their own matrices and an enamel tubule extension of the Tomes’ process is elaborated by the ameloblast in the same way that the odontoblast process extends during growth: this is what happens in proper tubular enamel in marsupials and other mammals—So why not in human enamel?

## Supplementary Information

Below is the link to the electronic supplementary material.

Supplementary Material 1
